# Recurrent, truncating *SOX9* mutations are associated with SOX9 overexpression, *KRAS* mutation, and *TP53* wild type status in colorectal carcinoma

**DOI:** 10.18632/oncotarget.9682

**Published:** 2016-05-29

**Authors:** Breanna M. Javier, Rona Yaeger, Lu Wang, Francisco Sanchez-Vega, Ahmet Zehir, Sumit Middha, Justyna Sadowska, Efsevia Vakiani, Jinru Shia, David Klimstra, Marc Ladanyi, Christine A. Iacobuzio-Donahue, Jaclyn F. Hechtman

**Affiliations:** ^1^ Department of Pathology, Memorial Sloan Kettering Cancer Center, New York, NY, USA; ^2^ Human Oncology and Pathogenesis Program, Memorial Sloan Kettering Cancer Center, New York, NY, USA; ^3^ Department of Medicine, Memorial Sloan Kettering Cancer Center, New York, NY, USA

**Keywords:** SOX9, colorectal carcinoma, KRAS, TP53, oncogene, Pathology Section

## Abstract

**Purpose:**

The extent to which the developmental transcription factor *SOX9* functions as an oncogene or tumor suppressor in colorectal carcinoma (CRC) is debatable. We aimed to clarify the effect of *SOX9* mutations on SOX9 protein expression and their association with known molecular subtypes and clinical characteristics in advanced CRC.

**Experimental Design:**

Next generation sequencing data (MSK-IMPACT) from CRC patients was used to interrogate *SOX9*, *KRAS, NRAS, BRAF, TP53, APC*, and *PIK3CA*. Mutant and wild type (WT) *SOX9* cases underwent immunohistochemical (IHC) staining to assess protein expression. *SOX9* allele-specific copy number was assessed by Affymetrix Oncoscan array.

**Results:**

*SOX9* was mutated in 38 of 353 (10.7%) CRC, of which 82% were frameshift or nonsense. Compared to *SOX9* WT, *SOX9* mutation was strongly associated with coexistent mutant *KRAS* (*p*=0.0001) and WT *TP53* (*p*=0.0004). SOX9 was overexpressed in both *SOX9* mutant and WT CRC. Among *SOX9* mutants, the highest expression was noted for truncating exon 3 mutants (mean H scores 239±105 versus 147±119, *p* value=0.02). Further, *SOX9* truncating mutants with loss of the WT allele demonstrated protein overexpression indicating the WT protein was not required for protein stabilization.

**Conclusions:**

SOX9 is overexpressed in CRC, including those with recurrent distal truncating mutations. The latter has structural similarity to the oncogenic isoform MiniSOX9, which is distally truncated due to aberrant splicing. This information suggests that truncated *SOX9* has oncogenic features. *SOX9* mutations are highly enriched in *KRAS* mutant and *TP53* wild type CRC; and may provide a therapeutic target in approximately 11% of CRC.

## INTRODUCTION

*SOX9* [sex-determining region Y (SRY)-box 9 protein] is a transcription factor that regulates development under normal circumstances. *SOX9* and other members of the SOX family have conserved structural features including a high mobility group (HMG) box DNA-binding domain, as well as a transactivation domain. The range of functions that Sox proteins serve is broad, including maintaining stem cell features, restricting lineage, and directing terminal differentiation. Germline *SOX9* heterozygous inactivating missense and nonsense mutations result in campomelic dysplasia, a syndrome resulting in skeletal malformations, central nervous system dysfunction, as well as abnormalities in other organs [1–2].

In the context of cancer, *SOX9* has been classified as both tumor suppressor and oncogene depending on the study and type of cancer being investigated. Evidence suggesting its role as a tumor suppressor is derived from several tumor types. SOX9 has decreased expression in cervical carcinoma compared to normal cervical tissue, and *in vitro* inhibited cell growth and tumor formation when overexpressed [3]. In prostate cancer, *SOX9* overexpression has been show to decrease cell proliferation and increase apoptotic activity.[4]. By contrast, both decreased *SOX9* activity and oncogenic features of SOX9 have been implicated in colorectal carcinoma (CRC) [5–7].

Recently, exome sequencing has identified *SOX9* as a recurrently mutated gene in colorectal carcinoma.[8]. The spectrum of mutations, effect on protein expression, molecular correlates, and clinical features has not been characterized to date. Here, we characterize the spectrum of *SOX9* mutations, their molecular and clinical correlates, and their effect on SOX9 protein expression in comparison to *SOX9* wild type (WT) carcinoma and normal epithelium within the context of CRC.

## RESULTS

### Characteristics of *SOX9* mutations in CRC

**Figure 1:**
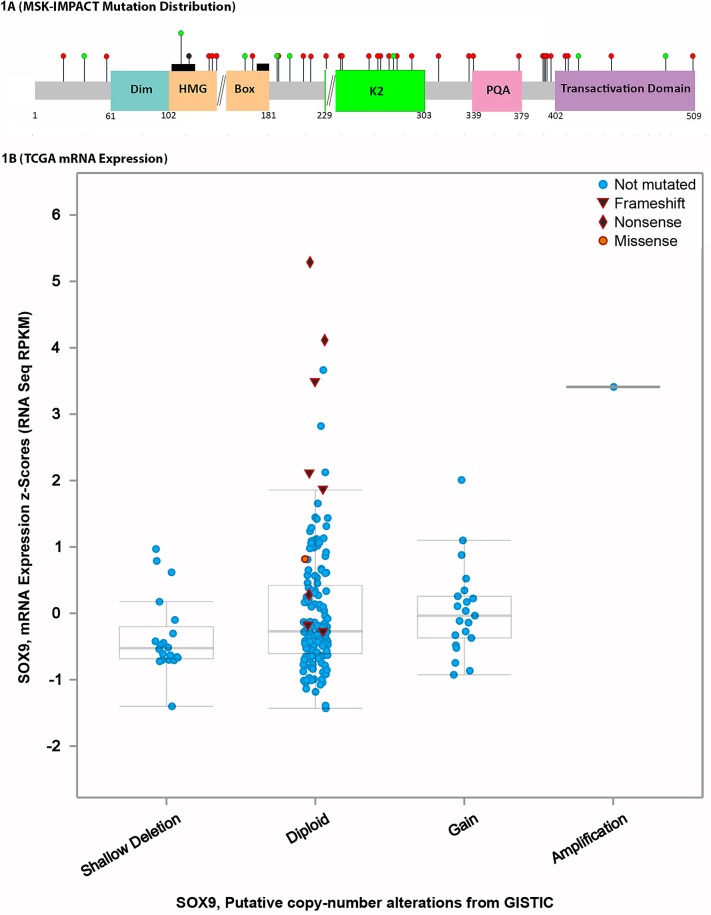
*SOX9* mutation distribution and transcription levels **A.** *SOX9*, which only has 3 exons (delineated by black double forward slashes), has a conserved DNA dimerization domain (Dim), a homeobox group binding domain (HMG box) with two nuclear localization signals (black rectangles) that bind DNA, and transactivation domains (K2 and Transactivation Domain) as well as a PQA domain. *SOX9* mutations occurred as mostly frameshift and nonsense mutations (red circles) while scattered missense mutations (green circles) and an in frame deletion (black circle) were also detected. The majority of mutations preserved the dimerization and HMG box domains. **B.** TCGA data shows that, similarly to our institutional study cases, most *SOX9* mutations are frameshift or nonsense. The mutations have increased RNA transcription levels on RNA sequencing.

Mutations discovered were either frameshift or nonsense mutations in 31 of 38 (81.6%) of *SOX9* mutant CRC. Each of the 38 *SOX9* mutations was unique, without recurrent mutations (Figure 1A, Table 1). There were 23 frameshift mutations, 12 nonsense mutations, 8 missense mutations, and 1 in frame deletion. The most frequently mutated exon was the last one (exon 3), which harbored predominantly nonsense and frameshift mutations (92%), while frameshift and truncating mutations only made up 44% of exon 1 mutation and 78% of exon 2 mutations (*p* = 0.02 for truncating mutations in exon 3 *versus* exons 1-2). There were 9 mutations in exon 1 (4 missense, 1 in frame deletion, 1 nonsense, and 3 frameshift), 9 mutations in exon 2 (2 missense mutations, 2 nonsense mutations, and 5 frameshift mutations) and 23 mutations in exon 3 (2 missense mutations, 9 nonsense mutations, and 15 frameshift mutations). TCGA data was similar to institutional data in that the majority of *SOX9* mutations detected were frameshift or nonsense. Further, the *SOX9* mutant CRC in TCGA data displayed transcriptional upregulation of SOX9 (Figure 1B).

**Table 1:** *SOX9* mutations, zygosity, and expression

Exon	Amino-acid change	Nucleotide change	Predicted AA length	TP (%)	AF (%)	H score (IHC)	Oncoscan result	Site Tested
1	E57Qfs*50	168_179delinsAG	107	22	45	300		liver
1	E134*	400G>T	134	10	11	0		primary
2	E159Gfs*25	474_475dupGG	184	20	21	60		peritoneum
2	E190*	568G>T	190	46	33	140		primary
2	Q208*	622C>T	208	25	50	0		primary
2	S215Pfs*4	643delT	219	11	7	300		primary
2	E191Rfs*28	571delG	219	18	24	300		peritoneum
2	Q164*, E226*	490C>T, 676G>T	226	50	10	130		primary
2	D168Efs*84	503dupA	252	48	39	70		liver
1	W143Lfs*109	427dupT	252	70	67	190	Gain, LOH	recurrence
3	V245Gfs*7	733dupG	252	50	25	300		liver
3	Q246Cfs*8	734_735dupTG	254	53	36	60		primary
3	L259*	776T>A	259	60	61	0		liver
3	P267Sfs*9	798_807delCCCTATCGAC	276	30	14	160		primary
3	G263Afs*16	788delG	279	25	13	1		primary
3	D274Wfs*6	818_819dupTG	280	40	29	300		primary
3	E277D, S279Afs*104	831G>C, 834delG	293	20	40			primary
3	Q312*	934C>T	312	30	82	300	Hypotriploid, LOH	ovary
3	W335*	1004G>A	335	30	76	300	Gain, no LOH	peritoneum
3	Q339*	1015C>T	339	10	25	300		liver
3	D290Mfs*93	868delG	383	50	44	300	Hypotetraploid, no LOH	primary
3	P374Rfs*9	1212C>A	383	20	53	300		primary, duodenum
1,2,3	S23FS, T196A, R394*	66delC, 586A>G, 1180C>T	394	30	15			primary
3	H396L, E400* in cis	1187A>T, 1198G>T	400	50	55	300	Gain, LOH	primary
3	Q393Sfs*10	1177delC	403	50	25	300		primary
3	H396Rfs*8	1185_1186dupGC	404	50	33	300		primary
3	Q410*	1228C>T	410	70	85	300	CN-LOH	primary
3	Q412*	1234C>T	412	50	73	225	CN-LOH	primary
3	H406Sfs*58	1215_1234del	464	60	23	300		primary
1	A118_A124del	353_373delCGGCGCGCAGGAAGCTCGCGG	502	54	21	300		primary
2	A187V	560C>T	509	60	25	5		primary
1	M113T	338T>C	509	90	28	90		primary
1	M113V, R162H	337A>G, 485 G>A	509	50	27	170		primary
1	S39C	116C>G	509	90	45			primary
3	V486A	1457T>C	509	25	20	250		recurrence
3	A419T	1255G>A	509	10	11	250		primary
3	R508_P509insGGL PRRAKMAEMILK ITEEREDQPEFPL DICVFLFFYFVLF FLLLLL*	1525_1530+5delCCTTGAGGAGG	557	10	18	0		primary
3	M469Ifs*109	1406dupT	578	50	54	3		liver

### Correlation of *SOX9* mutation frequency with known CRC genes

In comparison to *SOX9* wild type CRC, *SOX9* mutant CRC was strongly associated with the presence of *KRAS* mutation (*p* = 0.0001) and the absence of *TP53* mutation (*p* = 0.0004). *SOX9* mutant CRC also had higher rates of *PIK3CA* mutation (*p* = 0.0451), a trend toward *APC* mutation (*p* = 0.0527), and MMR deficiency (*p* = 0.0472). Interestingly, the majority of *SOX9* mutations in the MMR-D CRC were missense or nonsense and did not occur in mononucleotide tracts, while only 2 of the 10 SOX9 mutations in these 6 MMR-D, *SOX9* mutant CRC were frameshifts (both in mononucleotide tracts). None of the 38 SOX9 mutant CRC harbored *BRAF* or *NRAS* mutations, while 34 (10.8%) and 10 (3.2%) of *SOX9* WT CRC harbored *BRAF* and *NRAS* hotspot mutations, respectively (Table 2).

**Table 2:** Clinicopathologic and molecular characteristics of *SOX9* mutant *versus SOX9* WT CRC

	*SOX9* mutant (*n* = 38)	*SOX9* WT (*n* = 315)	*P* value
**Age (mean; median)**	60; 62	55; 59	
**Sex (M:F)**	22:16	184:131	
**Location: cecum to transverse**	18	107	
**Location: descending to rectum**	20	208	
**Histology: mucinous, poor**	18%, 0	4%, 10%	
**MMR-D**	26%	6%	*P* = 0.0472
***KRAS* mutant**	66% (25 of 38)	33% (123 of 315)	*P* = 0.0001
***NRAS* mutant**	0	11%	
***BRAF* mutant**	0	3%	
***APC* mutant**	87%	72%	
***TP53* mutant**	42% (16 of 38)	72% (227 of 315)	*P* = 0.0004
***PIK3CA* mutant**	32%	17%	*P* = 0.0451

### Correlation of *SOX9* mutation Type to immunohistochemical expression

**Figure 2:**
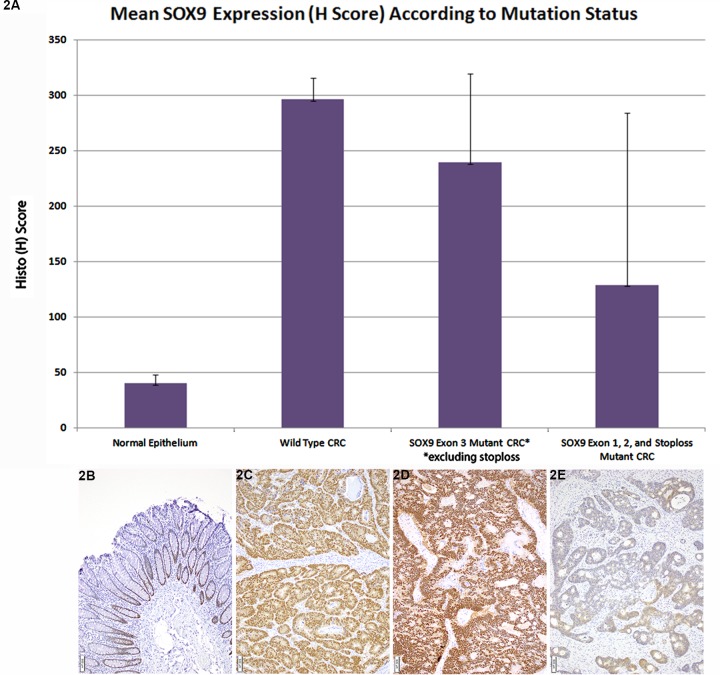
Mean SOX9 Expression (H Score) According to Mutation Status **A.** In comparison to normal epithelium (mean H score 40±7.9), which only shows SOX9 expression in deep crypt epithelium, the majority of both *SOX9* WT (mean H score = 296±19.2) and mutant CRC overexpressed SOX9. Among *SOX9* mutant CRC, truncating and missense exon 3 mutants had a higher mean H score (239±80.3) in comparison to other mutants including exon 1, exon 2, and exon 3 elongating mutants resulting in loss of stop codon (mean H score = 129±155.3), *p* = 0.02. **B.** Normal colorectal epithelium has nuclear SOX9 expression restricted to deep crypts. **C.** Strong, nuclear SOX9 expression is seen in *SOX9* wild type CRC. **D.** A *SOX9* p. Q312X mutant with loss of the normal allele displayed strong SOX9 nuclear expression. **E.** A *SOX9* frameshift mutants resulting in loss of the stop codon with paucity of nuclear SOX9 expression.

All 22 *SOX9* WT CRC analyzed showed strong positive expression (mean H-score 296±19.2). (Figure 2A, 2C) while normal epithelium shows weak expression limited to deep crypts (Figure 2A, 2B). Overall, *SOX9* mutant CRC showed a wide range of H scores with a mean H-score (188.7±123.3, *p* value < 0.001). However, closer review indicated that H scores in *SOX9* mutant CRC were highly related to both the type of variant and its site within the coding region of the *SOX9* gene. Truncating or missense exon 3 mutant CRC had higher H scores in comparison to other mutations (exon 1 or 2 mutant or exon 3 frameshift mutants resulting in loss of stop codon and predicted protein elongation) (*p* = 0.02): *SOX9* exon 1 or 2 mutant mean H score = 147, *SOX9* frameshifts resulting in loss of stop codon and predicted protein elongation had mutant mean H score = 2 (Figure 2A, 2D), exon 3 mutant CRC (excluding stop loss mutants) mean H score = 239 (Figure 2A, 2E).

### Overexpression of truncating *SOX9* mutations in the absence of a wild type allele

**Figure 3:**
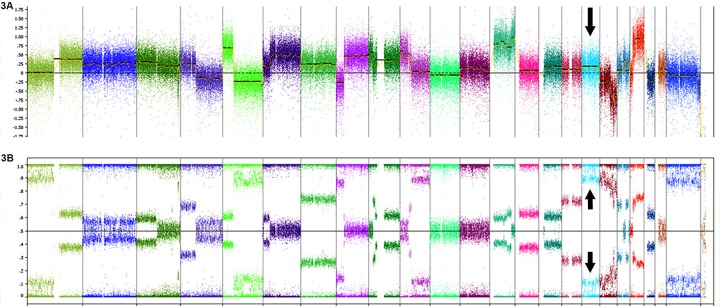
Copy number and heterozygosity of *SOX9* mutants **A.** Oncoscan analysis of the *SOX9* p. Q312X mutant displayed in Figure 2D shows gain of *SOX9* (arrow shows increase in log2 copy number over chromosome 17 including *SOX9*) and **B.** loss of normal allele (arrows show splitting of beta allele frequency).

Zygosity analysis was performed on 7 SOX9 expressing (IHC) CRC with *SOX9* frameshift or nonsense mutations and high *SOX9* mutant allele frequencies in comparison to estimated tumor percent. Five of these 7 cases showed LOH at chromosome 17q24.3 where *SOX9* is located. Of these 5 cases, 2 cases displayed copy neutral (CN) LOH while 3 harbored gains of the mutant allele (Table 1) in addition to LOH (Figure 3). The remaining two cases tested showed gain of the mutant allele yet retained the WT allele.

### Frequency of *SOX9* mutations in CRC and relationship to clinical features

**Figure 4:**
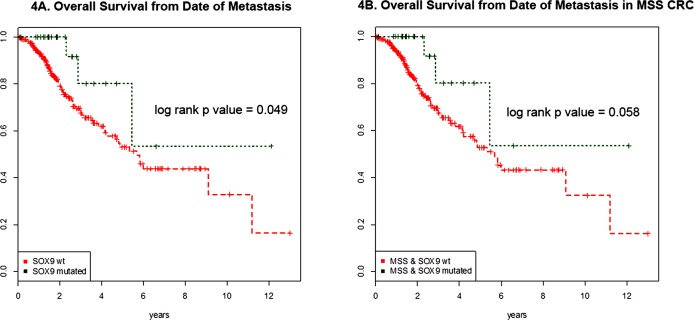
Survival curves of metastatic *SOX9* mutant and wild type CRC **A.** Overall survival from date of metastasis of *SOX9* mutant (green) and *SOX9* WT (red) are shown for the 317 CRC patients with metastatic disease (285 WT and 32 mutants) are shown. *SOX9* mutation was associated with better survival (log rank *p* value = 0.049) on univariate analysis. **B.** Adjusting for microsatellite instability, the overall survival of microsatellite stable (MSS) CRC are shown. This analysis excluded 2 microsatellite instability-high (MSI-H) *SOX9* mutant CRC and 30 MSI-H *SOX9* wild type CRC. MSS *SOX9* mutant CRC tended to have longer overall survival than *SOX9* wild type CRC from date of metastasis with borderline significance log rank *p* value of *p* = 0.058.

From January 1^st^ 2014 to December 31^st^, 2015; 353 CRC patients underwent MSK-IMPACT testing. Of these, 38 (10.7%) harbored *SOX9* mutations. *SOX9* mutant CRC did not differ from *SOX9* WT CRC in male to female ratio, patient age distribution, WHO morphologic subtype distribution, or primary tumor location (proximal *versus* distal) (Table 2). Patients with metastatic *SOX9* mutant CRC (*n* = 317) had longer overall survival in comparison to those with *SOX9* WT on univariate analysis (log rank *p* value = 0.049) (Figure 4A). To adjust for microsatellite status because MSI-H was more frequent in SOX9 mutant CRC, we compared overall survival in MSS CRC with *versus* without *SOX9* mutation. In this microsatellite stable analysis, 11 metastatic *SOX9* WT and 2 metastatic *SOX9* mutant CRC were MSI-H and excluded. Metastatic *SOX9* mutant CRC tended to have a longer overall survival of borderline statistical significance (log rank *p* value = 0.058) (Figure 4B).

Five patients with *SOX9* mutant, *RAS*/*RAF* WT CRC received anti-EGFR therapy (cetuximab or panitumumab) in combination with other treatment modalities (FOLFIRI, irinotecan). Of these, 3 (60%) showed progression of disease, 1 patient (unknown whether irinotecan naïve or not) received cetuximab and irinotecan and showed decreased tumor volume of hepatic metastasis, and 1 patient received adjuvant panitumumab with folfiri, floxuridine and a hepatic pump after metastatectomy and has not recurred.

## DISCUSSION

In this study, we demonstrate several findings including the recurrent nature of truncating SOX9 mutations in CRC, the overexpression of SOX9 in the majority of both *SOX9* mutant (including those with *SOX9* LOH) and WT CRC, and the strong association of *SOX9* mutation with mutant *KRAS* and WT *TP53*.

The distribution and nature of *SOX9* mutations in CRC is similar to that seen in classic tumor suppressors, namely mostly truncating mutations across the length of the gene without hotspots. However, the fact that we found gain of the mutant *SOX9* allele as well as overexpression of the mutant allele in the absence of the WT allele argues that truncated *SOX9* may serve as an oncogene in CRC, or as a gain of function tumor suppressor gene as well described in the case of *TP53*. However, data across the Cancer Genome Atlas (TCGA) show that *SOX9* is altered in a pattern typically seen in oncogenes: it is mutated and amplified in multiple cancer types, and rarely is the whole gene deleted [11]. The higher expression of SOX9 for exon 3 truncating mutations is potentially due to the fact that exon 3 is the last exon in *SOX9*, and truncating mutations within the last exon do not activate nonsense-mediated decay.[13]. This, however, may not be true for *SOX9* mutations that extend the protein *via* distal frameshift mutations, removing the WT stop codon and activating nonstop mediated decay [14] both of our cases with distal *SOX9* exon 3 frameshift mutations with stop loss mutations had very low H scores for expression (0 and 3 out of 300).

Interestingly, studies have shown overexpression of an isoform of the SOX9 protein in CRC that is truncated due to retention of intron 2 (termed ‘miniSOX9’), missing the transactivation domain yet still conserving the N terminal dimerization and DNA binding domains [15].

MiniSOX9 upregulates Wnt/β-catenin-dependent transcriptional activity and is associated with nuclear β catenin accumulation [16–18]. While one proposed explanation for this has been splice site mutations affecting intron 2, many CRC with miniSOX9 expression lack a mutational basis for the truncated and overexpressed SOX9 product. As the majority of *SOX9* mutations in CRC are truncating and result in deletion of the C-terminal protein including the transactivation domain, the protein product is predicted to be functionally similar to the MiniSOX9 isoform of SOX9.

The fact that truncated *SOX9* is highly associated with mutant *KRAS* and WT *TP53* has not been previously demonstrated. Wild type *SOX9* has been shown to cooperate with *RAS* mutants in several *in vivo* experiments [6] These experiments showed higher mRNA transcription levels of SOX9 when an *HRAS* codon 12 mutation was knocked in. These experiments also showed 5 times more tumor foci in a CRC cell line with *HRAS* codon 12 mutation when SOX9 was overexpressed in comparison to when SOX9 was not overexpressed [6] In regards to *TP53* status and correlation with *SOX9* mutation status, it has been found that overexpression of SOX9 results in lower levels of TP53 [6]. This indirect relationship may be due to SOX9 overexpression having decreased p14 levels, and p14 stabilizes TP53. Therefore, even though truncating *SOX9* mutations that result in SOX9 overexpression are associated with WT *TP53*, they may indirectly downregulate TP53 activity.

Finally, the correlation of *SOX9* mutation with longer survival has not been previously investigated. While we find that *SOX9* mutation is associated with increased survival in patients with metastatic CRC on univariate analysis (*p* = 0.049), we also know that *SOX9* mutation is associated with MMR deficiency in a non-random way as the majority of *SOX9* truncating mutations in MMR deficient CRC were not in mono- or di- nucleotide repeat areas. MMR deficiency has been shown to have a positive impact on prognosis [19]. Even after adjustment for microsatellite status, *SOX9* mutant MSS CRC tended to have longer overall survival than *SOX9* WT MSS CRC with borderline statistical significance (*p* = 0.058), suggesting that the *SOX9* mutation may positively affect prognosis independent of MSI status. A significant limitation within the survival data is that each patient's treatment cannot be compared or normalized for as there is a significant amount of variation.

In summary, we have provided evidence that truncating mutations in *SOX9* (particularly exon 3 truncating mutations) are recurrent in CRC and result in a truncated, overexpressed (in comparison to normal epithelium) protein that is likely oncogenic. These mutations are enriched in *KRAS* mutant, *TP53* WT cases; and are associated with better overall survival.

## MATERIALS AND METHODS

### Molecular testing

After approval by the local institutional review board, data from all patients with CRC being treated at Memorial Sloan Kettering Cancer Center between January 1, 2014 and December 31, 2015 who had molecular testing with Memorial Sloan Kettering- Integrated Molecular Profiling of Actionable Targets (MSK-IMPACT) was analyzed. MSK-IMPACT molecular testing is performed on MSKCC patients with CRC harboring distant metastases for *KRAS*, *NRAS*, and *BRAF* status analysis. This panel uses patient's matched normal and tumor DNA to interrogate somatic mutations, structural variants, and copy number alterations in all coding regions and select introns of 410 cancer-related genes as previously described in full detail (see reference) [9]. Mutations in the following genes were recorded: *KRAS* (c. G12, G13, Q61, A59, K117, A146), *NRAS* (c. G12, G13, A59, Q61, K117, A146), BRAF (c. V600E, fusions), *APC* (all coding mutations), *TP53* (all coding mutations), *PIK3CA* (all coding mutations) and *SOX9* (all coding mutations). Microsatellite instability (MSI) was assessed with MSIsensor [10] on next generation sequencing (NGS) data for cases that did not have mismatch repair (MMR) immunohistochemical (IHC) analysis for MLH1, PMS2, MSH2, and MSH6 expression retention (MMR-P) or loss (MMR-D). Tumor purity was estimated by a combination of histologic assessment and mutation allele frequencies. Possible candidates with *SOX9* loss of heterozygosity (LOH) included cases with mutant *SOX9* allele frequencies that were higher than half the estimated tumor purity. Cases with suspected *SOX9* LOH were studied for allele-specific copy number by Affymetrix Oncoscan arrays, provided they had sufficient remaining DNA.

Additionally, RNA transcription level data on *SOX9* mutant and WT CRC from The Cancer Genome Atlas (TCGA) was reviewed *via* cbioportal [11].

### Immunohistochemistry (IHC)

A cohort of 22 *SOX9* WT and all available (*n* = 35) *SOX9* mutant CRC underwent SOX9 IHC staining on whole sections of formalin fixed, paraffin embedded tissue. Immunohistochemical analysis for SOX9 expression was performed with the rabbit monoclonal SOX9 antibody, EPR14335-78 (Abcam, Cambridge, MA). Both the intensity of staining (0-3) and the percent of tumor cells staining (0-100%) were recorded for nuclear SOX9 IHC expression and used to generate a Histo (H) score that ranged from 0-300 [12]. Immunohistochemical overexpression of SOX9 was interpreted relative to normal colorectal epithelium.

### Clinical assessment

Electronic medical records were used to obtain data points including sex, age, tumor histology according to World Health Organization classification, primary site classified as either ‘proximal’ extending from cecum to distal transverse colon or ‘distal’ extending from splenic flexure to rectum, response to anti-EGFR therapy, time from metastasis to death were recorded.

### Statistical analysis

Statistical analysis was performed using Fisher's exact tests with two-tailed p values for categorical data, and unpaired Student's t tests for comparison of continuous data (IHC H scores). P values less than 0.05 are mentioned. P values less than 0.01 were considered statistically significant to approximately account for multiple hypothesis testing. Kaplan-Meier curves were drawn and a log-rank p-value was computed to compare survival from time of metastasis based on *SOX9* status for 317 patients who developed metastatic disease, including 285 *SOX9* WT and 32 *SOX9* mutant patients.
